# Pattern of changes in latissimus dorsi, gluteus maximus, internal oblique and transverse abdominus muscle thickness among individuals with sacroiliac joint dysfunction

**DOI:** 10.12669/pjms.35.3.62

**Published:** 2019

**Authors:** Muhammad Salman Bashir, Rabiya Noor, Mohammad Reza Hadian, Gholamreza Olyaei

**Affiliations:** 1 *Muhammad Salman Bashir, Ph.D, School of Rehabilitation, Tehran University of Medical Sciences, International Campus (IC-TUMS), Tehran, Iran*; 2 *Rabiya Noor, Ph.D, School of Rehabilitation, Tehran University of Medical Sciences, International Campus (IC-TUMS), Tehran, Iran*; 3 *Prof. Dr. Mohammad Reza Hadian, Ph.D, School of Rehabilitation, Tehran University of Medical Sciences, International Campus (IC-TUMS), Tehran, Iran*; 4 *Prof. Dr. Gholamreza Olyaei, Ph.D, School of Rehabilitation, Tehran University of Medical Sciences, International Campus (IC-TUMS), Tehran, Iran*

**Keywords:** Gluteal Maximus, LatissimusDorsi, Resting Thickness, Sacroiliac Joint, Ultrasound

## Abstract

**Background and Objective:**

Altered Pattern of the Global Muscle system is presented in literature among individuals with sacroiliac Joint Dysfunctions. However, the pattern of changes in the Latissimus dorsi (LD) and gluteal maximus (GM) among sacroiliac joint dysfunctions (SIJD) is not reported. This study aimed to investigate the changes in the resting muscle thickness of the Latissimusdorsi and gluteal maximus in SIJD.

**Method:**

A total of 88 subjects (44 individuals with SIJD and 44 healthy individuals as matched control) was included in this study. The resting thickness of the Latissimusdorsi and gluteal maximus was measured using real time musculoskeletal ultrasonography and data was compared between the ipsilateral side and contra lateral side among subjects with SIJD as well as healthy subjects. Independent sample t test was used to analyze the data by using SPSS version-25.

**Results:**

The results showed that contralateral LD were reduced significantly among subjects with SIJD when compared with the other side and with control. It also showed that ipsilateral IO, TrA and GM were reduced significantly among subjects with SIJD when compared with the controls and with contralateral side.

**Conclusion:**

The reduced resting muscle thickness showed an altered motor pattern of Deep Muscles of local system and Gross muscles of global system among patients with sacroiliac joint dysfunction.

## INTRODUCTION

Sacroiliac joint is a diarthrodial synovial joint and a source of low back pain and referred pain in the lower extremity.^1^ The sacroiliac joint has been shown to be a source of pain in 10% to 27% of suspected cases with chronic low back pain.^2^ Compressive forces across the pubic symphysis are increased by activation of the obliqus internal (IO) and adductor longus, whereas activation of the IO, transverses abdominus (TrA), gluteus maximus, latissimus dorsi, and lumbar erector spinae increase compressive forces across the SIJ.^3^ Mooney, Pozos, Vleeming, Gulick and Swenski have also used EMG to validate the relationship between gluteus maximus and latissimusdorsi on the SIJ. They found abnormal hyperactivity of the gluteus maximus on the involved side of SIJ and latissimus on the opposite side in subjects with symptomatic SIJ.^4^,^5^

The midpoint of the TrA muscle was found by measuring the horizontal length of the muscle. This midpoint was then used to measure the vertical distance between the upper and lower fascial lines of the TrA muscle to determine the muscle thickness. The thickness of the TrA muscle was measured at rest and with contraction. The average of the three trials was used for the calculations.^6^ The percent change of muscle thickness for the TrA was calculated using the following equation.^6^-^8^

Percent Change Thickness=(Contracted Thickness- Thickness at rest)/Thickness at rest *100^9^

### Diagnostic Tool

SIJ pain Provoking Test.^10^,^11^

Ultrasound is particularly useful because it is safe, noninvasive, and portable. Strong correlations have been reported between muscle thickness measured by B-mode ultrasound and site-matched skeletal muscle mass measured by MRI^12^-^16^ Therefore, it is plausible to use muscle thickness measurements to estimate muscle size and degree of muscle atrophy.^17^

Each measurement was repeated three times for rest, contracted and the mean used for calculation of Percent change Thickness.^18^,^19^ Measurements were obtained at the midline of the muscle belly and one cm to each side of midline The mean vertical distance of the 3 lines represented the muscle thickness value.^18^Although there is a general consensus that the GM becomes active after the Hamstring and Erector Spinae during the test there is some evidence that the onset of the GM is significantly delayed in LBP patients.^20^

Research question of this study was to find effect on contra lateral Latissimusdorsi and ipsilateral gluteus muscle. Transverse abdominus and internal oblique thickness in Sacroiliac Joint Dysfunction through ultrasonography. Thus it was hypothesized that Participants with Sacroiliac Joint Dysfunction may have reduced Resting thickness of Contra lateral LatissimusDorsi and Ipsilateral Gluteus Muscle, Transverse abdominus and Internal oblique. The findings of the study may help physiotherapists to design suitable exercise regime to deal with local and global muscle system among patients with sacroiliac Joint Dysfunctions (SIJD).

## METHODS

Study recruited 88 participants (n=44 participants (Opposite side and Ipsilateral) with SIJD and n=44 matched control). Participants were recruited on the basis of predefined selection criteria. Subjects with SIJD were selected from Gulabdevi Hospital and GanjBaksh Spinal & Research Rehabilitation and Life line Hospital. The inclusion criteria included three out of five provocative tests for Sacroiliac joint dysfunction namely Gaenslens, Thigh thrust, Sacral compression, Distraction, Faber test. The healthy subjects were recruited as controls from staff and primary care providers. The healthy subjects were matched as controls in terms of age, weight, height and body mass index (BMI). Any patients who reported disc pathology, history of spinal surgeries, any musculoskeletal symptoms on the lower limb over the past year and participants undergone any type of regular exercise over last three months were excluded. Informed written consent was obtained from participants after explaining the detailed procedure. The study was approved by the Research Ethics Committee of Tehran University of Medical Sciences-International Campus IR.TUMS.FNM.REC.1396.3668.

***Gluteus Maximus*** 30% proximal between posterior superior iliac spine and the greater trochanter.^17^ Measurement positions and measurement site for Gluteus maximus. Prone Lying 30% proximal between posterior superior iliac spine and the greater trochanter.^17^

***Position: For (TrA,IO)***

### Subject Position

Subjects were located on a base in crook lying by placing pillow below their head and the knees. Ultrasonic gel was medium between the transducer and the skin. The transducer was positioned in a transverse plane just higher to the right iliac crest along the axillary line.^18^,^19^,^21^ Place the transducer in the identical site during data collection carefully. At the end of exhalation, images were recorded.^18^,^19^

### Measurement Site

The Probe was placed on the anterolateral side of the abdominal wall, higher to the iliac crest and at right angles to the midaxillary line while ensuring that the center of the transversus abdominis muscle was centered within the field of sight.^18^ Each measurement was recorded three times for rest, contracted and the average used for calculation of Percent change Thickness.^18^,^19^ Dimensions were recorded at the central line of the muscle belly and 1 cm to each side of central line. The average perpendicular distanceof the 3 lines showed the muscle thickness value^18^All the muscle thickness measurements on the control participants were performed only on the dominant side. Each of the measurements was repeated three times and the mean was used to calculate the resting thickness. Prior to data collection, Intra-rater reliability of ultra-sound imaging measurements for resting thickness was measured. In pilot study assessor evaluated the reliability of ultrasound imaging of the deep abdominals (TrA,IO) and global muscles (GM,LD)in those with SIJ dysfunction and healthy matched controls. Three Measurements for LD, IO, TrA and GM were measured in a day after half an hour with 10 participants among which five were SIJD and five were healthy controlled matched. Results were highly consistent as ICC more than 0.90 showed the method was highly reliable.

## RESULTS

The mean (SD) of the age of the participants are shown in Table-I.

**Table-I:** Comparison of anthropometric findings.

Groups			

	Healthy Control	Sacroiliac Joint Dysfunction	p-value
Age	33.68±5.51	33.32±5.77	0.763
Weight	70.14±10.79	68.77±11.01	0.559
Height	5.55±0.34	5.56±0.33	0.902
BMI	24.66±4.36	24.10±4.39	0.548

Independent sample t-test

Resting Thickness of Latissimus Dorsi of Healthy Control was 13.04±2.25mm, among Sacroiliac Joint Dysfunction Ipsilateral was 12.12±1.67mm and opposite thickness was 8.69±1.13mm. Difference between thickness of healthy control from opposite (p-value <0.001) and Ipsilateral (p-value 0.039) was statistically significant. Similarly ipsilateral thickness was more than opposite side the difference was statistically significantly different from opposite (p-value <0.001).

Resting Thickness of transverse abdominus of Healthy Control was 4.02±0.14mm, among Sacroiliac Joint Dysfunction Ipsilateral was 3.95±0.17mm and opposite 3.48±0.10m thickness was. Difference between thickness of healthy control and Ipsilateral (p-value 0.039) was statistically significant. Similarly ipsilateral thickness was less than opposite side the difference was statistically significantly different from opposite (p-value <0.001).

Resting Thickness of Internal Oblique of Healthy Control was 8.78±0.89mm, among Sacroiliac Joint Dysfunction Ipsilateral was 5.75±0.52 mm and opposite thickness was 8.73±0.89mm. Difference between thickness of healthy control and Ipsilateral (p-value 0.039) was statistically significant. Similarly ipsilateral thickness was less than opposite side the difference was statistically significantly different from opposite (p-value <0.001)

Resting Thickness of gluteus Maximus of Healthy Control was 32.00±4.60mm, among Sacroiliac Joint Dysfunction Ipsilateral was 25.23±0.72mm and opposite thickness was 28.78±1.16mm. Difference between thickness of healthy control from opposite (p-value <0.001) and Ipsilateral (p-value <0.001) was statistically significant. Similarly ipsilateral thickness was less than opposite side the difference was statistically significantly different from opposite (p-value <0.001)

The mean (SD) of the resting thickness of the Latissimus dorsi and Gluteus Maximus between the ipsilateral side and contra lateral side of dysfunction are shown in the Table-II. The general trend from the results showed that the resting thickness of all the muscles (LD and GM) was smaller when compared with the opposite side among the participants. The results from paired test showed that the resting thickness for LD () and GM () was significantly smaller.All of the muscles showed a trend of reduced resting thickness among participants with SJD when compared with the healthy matched controls (Table-II).

**Table-II:** The mean (SD) of the resting thickness of the Latissimus dorsi, Gluteus Maximus, Transverse Abdominus and Internal oblique between the ipsilateral side and contra lateral side of dysfunction.

			Mean	Std. Deviation	Opposite	Ipsilateral
Resting Thickness of LatissimusDorsi	Healthy Control		13.04	2.25	<0.001	0.039
	Sacroiliac Joint Dysfunction	Opposite	8.69	1.13		<0.001
		Ipsilateral	12.12	1.67		
Resting Thickness of Transverse Abdominus	Healthy Control		4.02	0.14	0.070	<0.001
	Sacroiliac Joint Dysfunction	Opposite	3.95	0.17		<0.001
		Ipsilateral	3.48	0.10		
Resting Thickness of Internal Oblique	Healthy Control		8.78	0.89	0.494	<0.001
	Sacroiliac Joint Dysfunction	Opposite	8.73	0.87		<0.001
		Ipsilateral	5.75	0.52		
Resting Thickness of Gluteus Maximus	Healthy Control		32.00	4.60	<0.001	<0.001
	Sacroiliac Joint Dysfunction	Opposite	28.78	1.16		<0.001
		Ipsilateral	25.23	0.72		

**Fig.1:**
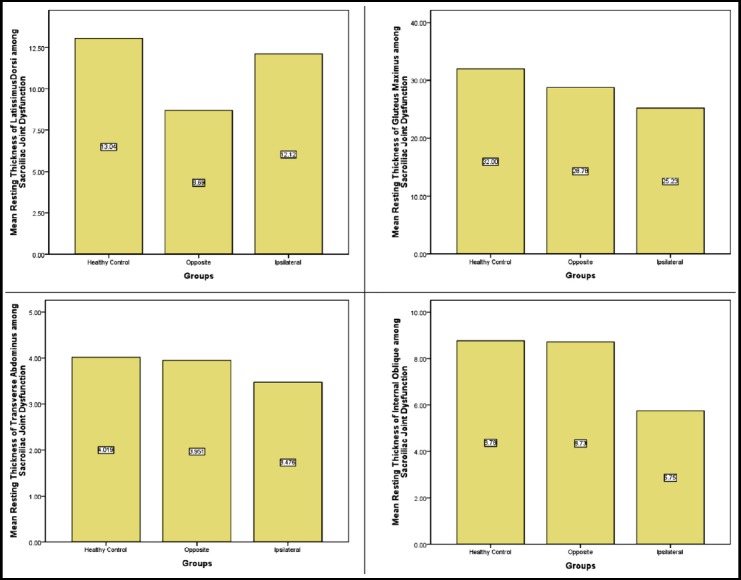
Mean resting thickness of latissimus dorsi, gluteus maximus, transverse abdominus and internal oblique.

## DISCUSSION

The current study investigated the changes in the resting thickness of the Deep muscles (TrA,IO) and Gross Muscles (LD,GM) among participants with SJD. The concept of testing the deep muscles and Gross muscles could be rationalized by the following evidence.^22^-^24^ The distortions of the pelvis as observed in SJD might occur secondary to the changes in pelvis and trunk muscle activity which might lead to directional strain and not positional changes within the sacroiliac joint.^22^ Such secondary changes mentioned in the pelvis and trunk muscle activity imply study of the LS and GS of the sacroiliac joint. Secondly, a study conducted using Doppler imaging of vibrations to examine laxity on the sacroiliac joint reported that the voluntary unilateral contractions of relevant muscles of the pelvis resulted in reduced mobility of the sacroiliac joint on the ipsilateral side 23. Thirdly, adequate compression of the pelvis joint surfaces was suggested as the result of re-action forces acting across the joint through muscle cocontractions and ligament tension^24^. In consideration to the above studies, it may be apparent that the LS and GS that cross the pelvic joint need to be studied for understanding the biomechanical alterations in SJD. In addition, the conceptual model of stability established by Panjabi^25^ explains the need to investigate the local and global system in SJD. As per this model, the lumbopelvic stability is maintained by the interaction between the passive, active, and control system.^25^

Therefore any excessive stress on the osteoarticular ligamentous passive system as it might be presented in SJD as likely to alter the proprioceptive input from the passive system to the control system. In turn, the resulting altered output from the control system might impair and alter the muscle thickness and contractility of the muscles that cross the sacroiliac joint. Therefore, in the current study, it was hypothesized that the muscle thickness of the Deep Muscles (TrA, IO) and Gross Muscles(LD, GM) might be reduced in size due to the altered control system in SJD.

All the LS(TrA & IO) and GS(GM) in this study that cross the sacroiliac joint showed a trend of reduced resting thickness of the muscles on the side of SJD when compared with the contralateral joint as well as against the matched healthy individuals. However, muscle thickness of LD reduced on contralateral side as compared to ipsilateral as well as against the matched healthy controls. Therefore, true significance was observed only in the resting muscle thickness of the IO, TrA and GM on the side of dysfunction when compared with the contra lateral side in the sacroiliac joint and compared with healthy individuals. The findings of the study imply that the LS and GS tend to be impaired and altered in SJD. The trend of reduced thickness of the LS and GS is supported by several past studies that also had reported delayed muscle activity of the LS and GS among patients with lumbar and pelvic girdle pain.^26^-^28^ The LS and GS work together to create a rigid cylinder of abdominal cavity there by protecting the mechanical stress to sacroiliac joint and aids in normal load transfer to the pelvis and lower extremities. The reduced thickness of the muscles might affect the biomechanical property of the joint by altering the mechanical stress and load transfer. Nevertheless with SJD reported to cause 22.5% of back pain, the altered biomechanical changes in the LS and GS may explain the role of SJD as one of the reasons for development of low back pain. Hence, clinicians might consider suggestions of an appropriate exercise program to train the LS and GS muscle system to manage lumbopelvic disorders. Another limitation of the study is that the effect of limb dominance on SJD was not explored in the current study but will be more fully investigated in a future study. Activities such as active straight leg raises were shown to activate and increase the thickness of IO, EO, and TrA muscles.^28^

Perhaps, clinicians might use active straight leg raises and Prone Hip extension as a therapeutic movement to strengthen the core stability among individuals with lumbopelvic disorders where LS and GS were compromised.^29^

### Limitations of the study

The measurement of the thickness of the LS and GS during rest is one of the limitations of the study. The muscles are not assessed during contraction or during any functional tasks related with sacroiliac joint which might be more appropriate for the functional role of the joint and the muscle system.

## CONCLUSION

The reduced resting muscle thickness shows an altered motor pattern of Deep Muscles of local system and Gross muscles of global system among patients with sacroiliac joint dysfunction. Future studies should consider examining the biomechanical effects of altered LS and GS in SJD by looking into functional tasks such as prone hip extension, Active straight leg raise and load transfer during gait.
